# Genome-wide identification of miRNAs and their targets during early somatic embryogenesis in *Dimocarpus longan* Lour.

**DOI:** 10.1038/s41598-020-60946-y

**Published:** 2020-03-13

**Authors:** Xiaoping Xu, Xiaohui Chen, Yan Chen, Qinglin Zhang, Liyao Su, Xu Chen, Yukun Chen, Zihao Zhang, Yuling Lin, Zhongxiong Lai

**Affiliations:** Institute of Horticultural Biotechnology, Fujian Agriculture and Forestry University, Fuzhou, 350002 China

**Keywords:** Biological techniques, Biotechnology, Cell biology, Computational biology and bioinformatics, Developmental biology, Molecular biology, Plant sciences, Stem cells

## Abstract

miRNAs are endogenous regulatory factors that play pivotal roles in post-transcriptional regulation. However, their specific roles in early somatic embryogenesis (SE) remain unclear. Study of the SE system is fundamental for clarifying the molecular mechanisms in *Dimocarpus longan*. We identified 289 known miRNAs from 106 different miRNA families and 1087 novel miRNAs during early longan SE, including embryogenic callus (EC), incomplete pro-embryogenic culture (ICpEC), globular embryo (GE), and non-embryogenic callus (NEC). The abundances of known miRNAs were concentrated in GE. The differentially expression (DE) miRNAs showed five expression patterns during early SE. Largely miRNAs were expressed highly and specially in EC, ICpEC, and GE, respectively. Some miRNAs and putative target genes were enriched in lignin metabolism. Most potential targets were related to the pathways of plant hormone signal transduction, alternative splicing, tyrosine metabolism and sulfur metabolism in early longan SE. The regulatory relationships between dlo-miR166a-3p and *DlHD-zip8*, dlo-miR397a and *DlLAC7*, dlo-miR408-3p and *DlLAC12* were confirmed by RNA ligase-mediated rapid amplification of cDNA ends. The expression patterns of eight DE miRNAs detected by qRT-PCR were consistent with RNA-seq. Finally, the miRNA regulatory network in early SE was constructed, which provided new insight into molecular mechanism of early SE in longan.

## Introduction

Plant somatic embryogenesis (SE) is physiologically and morphologically similar to that of zygotic embryos. Therefore, SE is usually used as a model system for studying embryonic development in higher plants. Previously study demonstrated that transcription factors (TFs) regulated embryogenic transformation in SE^1^; this TFs included *BABY BOOM (BBM)*, *AGAMOUS-LIKE15* (*AGL15*), *LEC2* and *SERK*, which functioned in the process of cell division and differentiation in embryonic development^2^. miRNAs are a kind of small endogenous RNAs of 18–24nt in length, which mainly recognize the complementary binding sites of their target mRNAs. miRNAs were transcribed by RNA polymerase II (Pol II). miRNAs could mediate mRNA cleavage and inhibiting translation at transcriptional or post-transcriptional level in eukaryotes^3,4^. Over the past decade, miRNAs have been reported to be involved in a broad range of metabolic and physiological processes in plants, such as plant growth, development and responses to stresses. However, the regulation of miRNA of plant SE was still unclear.

The miRNA regulatory pathway is particularly important in plant growth and development, hormone regulation, organ differentiation, alternative splicing and secondary metabolite accumulation^5–7^. The SE could be influenced by epigenetics in some plant^8^. For instance, expression levels of miR171, 159, 169 and 172 affected in the embryogenic potency of *Larix gmelini*^9^, these particular miRNAs targeted to multifunctional TFs and participated in ABA signal transduction, growth and development, and stress response. In *Citrus sinensis* (L.), miR156, 168 and 171 regulated the early development processes of SE. Besides, miR159, 164, 390 and 397 cooperatively regulate formation of the globular embryo. miR164, 166 and 397 could regulate the cell lines… which lack embryogenesis potential ability^10^. Lin and Lai^11^ analysed the miRNAs in different stages of embryonic development in longan. In addition, the miRNAs were also expressed in embryos such as spruce^12^, rice^13^, grape^14^, cotton^15^, wheat^16^ and lily^17^. However, most of these studies mainly focused on sequencing of the miRNA transcriptome at certain embryonic stages or in mixed samples, without referencing the genome of that species. This was likely to decrease the accuracy of miRNA transcriptional sequencing in species-specific stages of embryonic development.

Longan (*Dimocarpus longan* Lour.), a member of the Sapindaceae family, is an economically important subtropical evergreen fruit tree in China. Recent studies indicated that seed abortion normally occured during early embryonic stage in this species^18–20^, but it was a challenge to collect raw materials from early-stage zygotic embryos in order to study this. Consequently, it was important to clarify the molecular mechanisms involved in SE development in longan. Lin^21^ revealed some miRNAs were stably expressed during the development of SE. The functions of the miRNAs (miR398, 393, 160 and 390) were further verified and analysed during the maturation of SE in longan^22–24^. The findings revealed that the miRNA regulatory network played an important role in the development of SE in longan, and early somatic embryo development was closely associated with the totipotency of differentiated embryogenic calli and the seed size.

To separate miRNA from early SE in longan, in this study, miRNAs and their target genes were identified from the early SE (embryogenic callus, EC; incomplete embryogenic compact structure, ICpEC; and globular embryo, GE) and non-embryogenic callus (NEC). The differential expression patterns and functional enrichment of metabolic pathways of miRNAs were analysed, and the essential roles of miRNAs in early SE were discussed. The results of this study provided insight into the specific miRNA regulatory network in early SE of longan.

## Methods

### Plant materials and RNA isolation

The somatic embryogenesis system of ‘Honghezi’ longan was constructed by Lai Zhongxiong^25–27^. The materials from four different stages of somatic embryogenesis were obtained in longan: embryogenic callus (EC), incomplete pro-embryogenic culture (ICpEC), globular embryo (GE) and non-embryogenic callus (NEC). EC, ICpEC and GE were obtained by culturing on MS + 1.0 mg/L 2,4-D, MS + 0.5 mg/L 2,4-D and MS + 0.1 mg/L 2,4-D, respectively, for 20d. The cellular morphology of EC was loosely packed and pale yellow, ICpEC was more tight, and that of GE was more tightly packed and featured protoderm cells. NEC was obtained by culturing on MS + 4.0 mg/L 2,4-D for 45 d^27^, and showed an irregular cell shape. After freezing in liquid nitrogen, the samples were stored at −80 °C for the extraction of total RNA for RNA-seq and quantitative reverse-transcriptase polymerase chain reaction (qRT-PCR).

Total RNA of the materials in the four stages was extracted with TRIZOL Reagent (Invitrogen, USA), in accordance with the manufacturer’s instructions. Materials from all stages were analysed as three biological replicates. One percent agarose gel electrophoresis and an ultramicro-ultraviolet spectrophotometer were used to determine the quality of RNA. The RNA samples with A_260_/A_280_ ratios between 1.9 and 2.1 and A_260_/A_230_> 2.0 were used for further RNA ligase-mediated rapid amplification of cDNA ends (RLM-RACE) PCR and qRT-PCR analyses.

### Small RNA library construction and HiSeq sequencing

The four small RNA libraries (EC, ICpEC, GE, NEC) of longan were constructed and sequenced using the Illumina HiSeq2000.platform (Beijing Genome Institute, BGI). First, the electrophoretic bands of RNA between 18 and 30nt were separated on a PAGE gel and small RNA was recovered. A 5′ adapter was connected to the small RNA by T4 RNA ligase, followed by mixing, centrifugation and reaction at a suitable temperature for the set time. PAGE gel was used to purify and recover the 5′ ligation product. The method to purify and recover the 3′ ligation product was the same as for preparation of the 5′ ligation product. Second, Superscript II reverse transcriptase (Invitrogen) was used to synthesise the first strand of cDNA; the reverse transcription primer was 5′-CAAGCAGAAGACGGCAGCACGA-3′. Third, the small RNA libraries were constructed by bridging PCR and PAGE gel. Finally, Agilent 2100 Bioanalyzer^28^and the ABI Step One Plus Real-Time PCR System were used to detect the high throughput and accuracy of the smallRNA libraries.

### miRNA identification and bioinformatic analysis

After removing the low-quality reads, such as those containing poly-N, 5′ adapter contaminants, no 3′ adapter or insert tag, or those containing poly(A), (T), (G) or (C), from the raw data, the clean data were obtained. The clean tags were mapped to the longan genome (NCBI accession number: BioProject PRJNA305337)^29^ and longan embryogenic callus transcriptional database (SRA050205)^30^ by SOAP or Bowtie^31^. The expression and distribution of sRNA in the genome were analysed. Second, the quality, length, and public sRNA sequences were analysed statistically. Noncoding RNA, such as miRNA, siRNA, piRNA, rRNA, tRNA, snRNA and snoRNA, was identified via comparisons with miRBase21.0 (http://www.mirbase.org/ftp.shtml), Rfam (11.0)^32^ and GenBank (http://www.ncbi.nlm.nih.gov/genbank/); the precursor sequences of potential miRNA were extracted and predicted with miREAP software (https://sourceforge.net/projects/mireap/) by comparison with known miRNAs in miRBase 21.0. Then, the known miRNAs were determined by comparing with the known database alignment and conserved miRNA sequences. Mireap and Mfold were used to annotate the sRNAs and make a single annotation for each unique sRNA. sRNA was traversed according to the priority order of rRNA> known miRNA> piRNA> repeat> exon> intron. Then compared exon antisense chain, intron and intergenic region^33,34^, if they did not annotate and map to the longan genome then they would be used to predict the novel miRNA by Mireap (a software independently developed by HuaDa for predicting miRNAs in plants and animals), and the novel secondary structure map was plotted. Finally, the first base distribution of candidate specific miRNAs was analysed statistically and the accuracy of the predicted results was judged. Gene Ontology (GO) functional annotation^35^ and Kyoto Encyclopedia of Genes and Genomes (KEGG) pathway analyses (www.kegg.jp/kegg/kegg1.html)^36^ were used to investigate the functions of the target genes.

### Differential expression analysis of miRNAs

The differential expression of known and novel miRNAs (DE miRNAs) was normalised in the four libraries using the ExpDiff method (Shenzhen, China). The miRNAs in the four libraries were normalised to one million to reduce potential errors before calculating the fold change, *p*-value and ratio as follows^37^: Normalised expression = (actual miRNA count / total count of clean reads) × 1,000,000; Fold change = log2 (miRNA normalised read count in the treatment library / miRNA normalised read count in the control library). The *p*-value was calculated as follows:

$${\rm{p}}({\rm{y}}/{\rm{x}})=(\frac{{N}2}{{N}1})y\frac{({\rm{x}}+{\rm{y}})!}{{\rm{x}}!{\rm{y}}!(1+\frac{{N}2}{{N}1})({\rm{x}}+{\rm{y}}+1)}\begin{array}{ccc}{C}({\rm{y}}\le {{\rm{y}}}_{\min }|{\rm{X}}) & = & \mathop{\sum }\limits_{{\rm{y}}=0}^{{\rm{y}}\le {{\rm{y}}}_{\min }}{\rm{p}}({\rm{y}}|{\rm{x}})\\ {D}({\rm{y}}\le {{\rm{y}}}_{\max }|{\rm{X}}) & = & \mathop{\sum }\limits_{{\rm{y}}={{\rm{y}}}_{\max }}^{\infty }{\rm{p}}({\rm{y}}|{\rm{x}})\end{array}$$

In the formula, N1 and N2 represent the total counts of clean reads in two sRNA libraries, and x and y stand for the counts of miRNAs in the two sRNA libraries. The thresholds of log2 (fold change)|>1 and *p*-value < 0.01 were applied to define differential expression of the miRNAs between the two libraries, under the same circumstances, the *p*-value < 0.05 suggested that the difference was significant. In this study, the ratio defined by fold change revealed a miRNA expression of one stage of SE library compared with that in another stage library. A ratio> 2 meant the miRNA was upregulated, and a ratio < 1/2 meant the miRNA was downregulated. Standardised results were used to count fold_change, *p*-value and made drawings^37^. The DE miRNAs of different stages were identified using a Venn diagram (http://bioinformatics.psb.ugent.be/webtools/Venn/).

### Prediction and annotation of the target genes of miRNAs

psRNATarget^38^ was used to identify the relationship between potential miRNAs and their target genes. Then, in the NR database, Blastx with an e-value less than e-10 was used to search the candidate targets to predict their possible functions. The functions of the differentially expressed miRNAs were analysed using Blast2GO software^39^.

### Modified 5′ RLM-RACE and real-time quantitative PCR

psRNATarget was used to predict the relationships of dlo-miR397a and *DlLAC7*, dlo-miR166a-3p and *DlHD-zip*, dlo-miR408-3p and *DlLAC12*. Gene Racer Kit (Invitrogen) reverse-transcription kit was used to synthesise cDNA template GeneRacer 5′ primers and gene-specific primers were designed according to the target gene sequence and miRNA matching position (Supplemental Data S1). The PCR products were purified, cloned and sequenced. The annealing temperatures of the first- and second-round PCR of cleavage sites validated PCR procedure were 57 °C and 56 °C, and the extension time was 30 s. The GeneRacer™ 5′ primer was 5′-CGACUGGAGCACGAGGACACUGA-3′ and GeneRacer 5′ nested primer was 5′-GGACACUGACAUGGACUGAAGGAGUAGAAA-3′, which were used as universal primers.

The first-chain cDNA of miRNA qRT-PCR was synthesised by poly(A) miRNA-based qRT-PCR in accordance with the TransScript miRNA First-Strand cDNA Synthesis SuperMix Instruction Manual; then, the cDNA was diluted tenfold and an miRNA-qRT-PCR reaction system was established consisting of 0.4 μL of forward primer, 0.4 μL of universal primer, 10 μL of 2 × TransStart Tip Green qPCR SuperMix, 2 μL of cDNA template and 7.2 μL of ddH_2_O. The procedure was as follows: predenaturation at 95 °C for 30 s, followed by 40 cycles of denaturation at 95 °C for 10 s, annealing at 60 °C for 30 s and extension at 72 °C for 15 s. The first-chain cDNA of genes was synthesised by qRT-PCR using the PrimeScript RT Reagent Kit (Takara), in line with the instruction manual. cDNA from the three stages of somatic embryogenesis was diluted tenfold. The qRT-PCR reaction system consisted of 10 μL of SYBR, 2 μL of cDNA template, 0.8 μL each of forward and reverse primers, and 6.4 μL of ddH_2_O. The procedure was as follows: initial denaturation for 30 s at 95 °C, followed by 40 cycles of denaturation at 95 °C for 10 s, annealing at 60 °C for 30 s and extension at 72 °C for 15 s, performed on a LightCycler 480 instrument (Roche Applied Science, Switzerland). Each reaction was performed in triplicate. cDNA was diluted into different concentrations of 5×, 25×, 125× and 625× to make a solution curve. *DlU6*^21^ and *DlUBQ*^40^ were used as internal controls for the miRNAs and their potential target genes, respectively. Three biological and three technical replicates were used for analysing the expression of each miRNA and their potential target genes. The primers of miRNAs and their potential targets were used in qRT-PCR analyses (Supplemental Data S1). The expression level of miRNAs and their potential targets were calculated by the 2^−ΔΔCt^ method. Finally, one-way ANOVA was performed in SPSS (Statistical Product and Service Solutions) to assess the statistical significance of differences in the data.

## Results

### The categories and sizes of sRNA of longan early SE varied greatly

Illumina HiSeq.used to construct a small RNA database of the four stages (EC, ICpEC, GE, and NEC). A total of 47,791,087 raw reads were generated. Clean reads comprised more than 90% of raw reads (Supplemental Data S2). In total, 80.78%–86.19% of the reads were perfectly matched to the longan genome (Table 1). Overall, 7099 (0.7%) to 10658 (0.26%) unique miRNA sequences were obtained in the four stages. The number of miRNAs in early SE was more than in NEC, and markedly larger in GE (Table 1). In addition, a total of 289 known mature miRNAs were identified, belonging to 106 miRNA gene families. In total, 140, 168, 163 and 116 mature miRNAs were identified in EC, ICpEC, GE and NEC, respectively (Supplemental Data S3). Most families had only one member; some families had two members, such as miR157, 159, 162, 168, 169, 319, 390, 393, 394, 395, 397, 408, 2592 and 396. Furthermore, miR159, 171 and 166 included five members; miR156 and 482 included six members; and miR167, 2592, 2118, 398 and 827 were consisted of three members respectively (Supplemental Data S4).

**Table 1:** Distribution of total and unique small RNA (sRNA) sequences from the early SE (EC, ICpEC, GE) and NEC. “Unann” refers to sRNA sequences for which no annotation information by database comparisons.

Types	Unique				Total			
	NEC	EC	ICpEC	GE	NEC	EC	ICpEC	GE
Total sRNAs	1008620 (100%)	3116033 (100%)	4069330 (100%)	4102483 (100%)	10737344 (100%)	11439171 (100%)	11086034 (100%)	11870025 (100%)
Mapping to genome	703592 (69.76%)	2304585 (73.96%)	2995773 (73.62%)	2902693 (70.75%)	9169172 (85.4%)	9859682 (86.19%)	9068010 (81.8%)	9588866 (80.78%)
exon_antisense	33304 (3.3%)	86317 (2.77%)	118027 (2.9%)	110712 (2.7%)	1058371 (9.86%)	899353 (7.86%)	597904 (5.39%)	679814 (5.73%)
exon_sense	73712 (7.31%)	198704 (6.38%)	177847 (4.37%)	171952 (4.19%)	1304559 (12.15%)	1333048 (11.65%)	791515 (7.14%)	939647 (7.92%)
intron_antisense	27405 (2.72%)	72938 (2.34%)	102814 (2.53%)	117463 (2.86%)	629664 (5.86%)	690305 (6.03%)	433831 (3.91%)	559132 (4.71%)
intron_sense	35807 (3.55%)	99419 (3.19%)	134189 (3.3%)	147545 (3.6%)	728128 (6.78%)	653364 (5.71%)	518287 (4.68%)	610314 (5.14%)
miRNA	7099 (0.7%)	8905 (0.29%)	8830 (0.22%)	10658 (0.26%)	1398392 (13.02%)	421499 (3.68%)	354236 (3.2%)	403510 (3.4%)
rRNAs	34742 (3.44%)	41221 (1.32%)	28582 (0.7%)	36052 (0.88%)	1794585 (16.71%)	1735104 (15.17%)	642321 (5.79%)	984229 (8.29%)
repeat	77282 (7.66%)	481696 (15.46%)	639164 (15.71%)	479149 (11.68%)	393265 (3.66%)	1242134 (10.86%)	1769925 (15.97%)	1279780 (10.78%)
snRNAs	2246 (0.22%)	2873 (0.09%)	2028 (0.05%)	2883 (0.07%)	62387 (0.58%)	43714 (0.38%)	21565 (0.19%)	43480 (0.37%)
snoRNA	2212 (0.22%)	3356 (0.11%)	2504 (0.06%)	3774 (0.09%)	57337 (0.53%)	84727 (0.74%)	38772 (0.35%)	92584 (0.78%)
tRNA	4251 (0.42%)	7064 (0.23%)	4720 (0.12%)	5896 (0.14%)	119579 (1.11%)	122075 (1.07%)	61901 (0.56%)	80031 (0.67%)
unannnotated	710560 (70.45%)	2113540 (67.83%)	2850625 (70.05%)	3016399 (73.53%)	3191077 (29.72%)	4213848 (36.84%)	5855777 (52.82%)	6197504 (52.21%)

**Figure 1:**
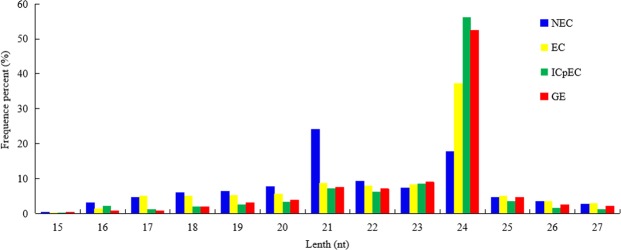
Size distributions of miRNAs in the early SE in longan.

**Figure 2:**
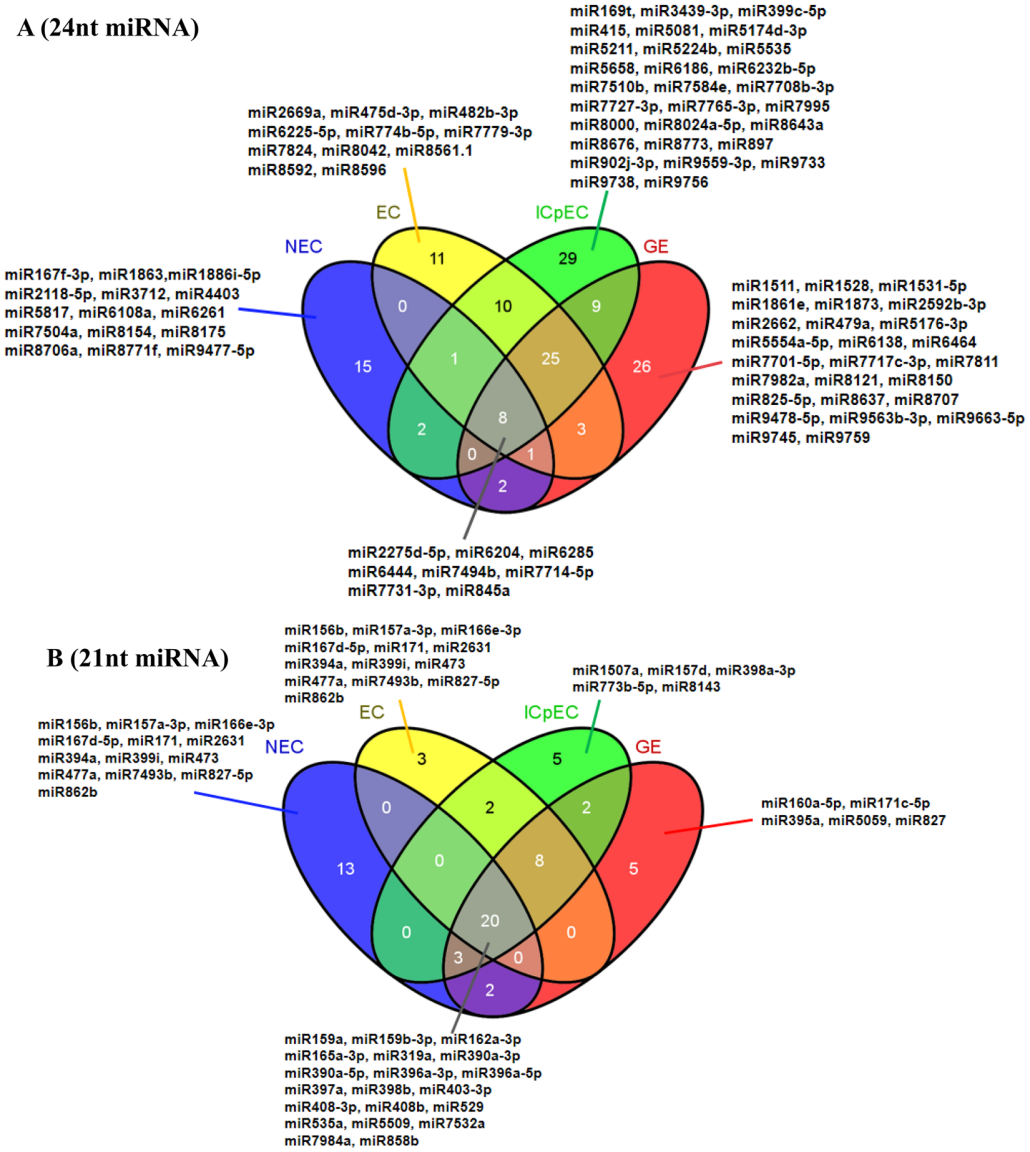
Quantitative distribution of 24nt, 21nt miRNA in the early SE and NEC of longan. (**A**) represents the distribution of 24nt miRNA at different stages of longan; (**B**) represents the distribution of 21nt miRNA at different stages of longan.

In general, the size of sRNAs ranged from 18 to 30nt in the four stages (EC, ICpEC, GE, and NEC). The most common sizes of miRNAs were 24 and 21nt. The development of early SE was mainly regulated by 24nt miRNAs, NEC was mainly regulated by 21nt miRNAs. The distributions of small RNA categories and their sizes were shown in Fig. 1. In total, 142 24nt miRNAs of four stages, 15 24nt miRNA were specifically expressed in NEC; 25 24nt miRNA were co-expressed in the three stages of EC, ICpEC and GE (Fig. 2A). A total of 63 21nt miRNAs were identified in four stages, 13 of them were expressed in NEC, while 8 of them were in EC, ICpEC and GE. The results showed that 20 miRNAs were co-expressed in the four stages (Fig. 2B). The numbers of 19, 20 and 22nt miRNAs were much fewer than those of 24nt and 21nt miRNAs (Supplemental Data S5).

### Identification of known and novel miRNAs during early SE in longan

To identify the known conserved miRNAs, sRNAs were compared with other annotated miRNAs of plants in the miRBase database by Blast or Bowtie. Overall, 110, 118, 118, and 101 conserved miRNAs were found in EC, ICpEC, GE, and NEC, respectively. In addition, in these groups, 213, 398, 381, and 612 novel hairpin sequences (Supplemental Data S6) and 147, 245, 251, and 394 novel mature miRNAs were predicted and obtained (Supplemental Data S7). Moreover, 1250, 2438, 2482, and 3543 target genes were regulated by the novel mature miRNAs, with 1308, 2754, 3055 and 4042 targeted cleavage sites. The novel mature miRNAs ranged from 20 to 23nt in length, the most common sizes were 21 and 22nt in NEC, while the main size was 23nt miRNA in ICpEC (Supplemental Data S7).

In addition, the mature miRNA was cleaved by dicer, and the first specificity base site was preferentially U (uracil). In our study, when the predicted novel miRNA cut its target gene, the 10th shear site tended to A. The percentages of adenine (A), uracil (U), guanine (G) and cytosine (C) of the miRNA site were predicted in the changed dynamically development of longan early SE. Based on the analysis of the first base bias of 20–23nt novel miRNA showed that (Supplemental Data S8, Fig. S1 NEC–GE), the first base of 23nt miRNAs were all biased to U in the four stages, the 20nt miRNAs were not showed a regular rule. In addition, the results of novel miRNA nucleotide bias at each position analysis showed that (Supplemental Data S8, Fig. S1 NEC–GE): in early SE (EC, ICpEC, GE), the first base was preferentially U and the 10th was prefer to A or G, this consistent with the general rule of miRNA base distribution. At the NEC stage, the first base of miRNA was preferentially C rather than U, and the 10th base was preferentially G rather than A. The above results were inconsistent with the general rule of base distribution of miRNA.

### Differentially expressed miRNAs durimg early SE in longan

**Figure 3:**
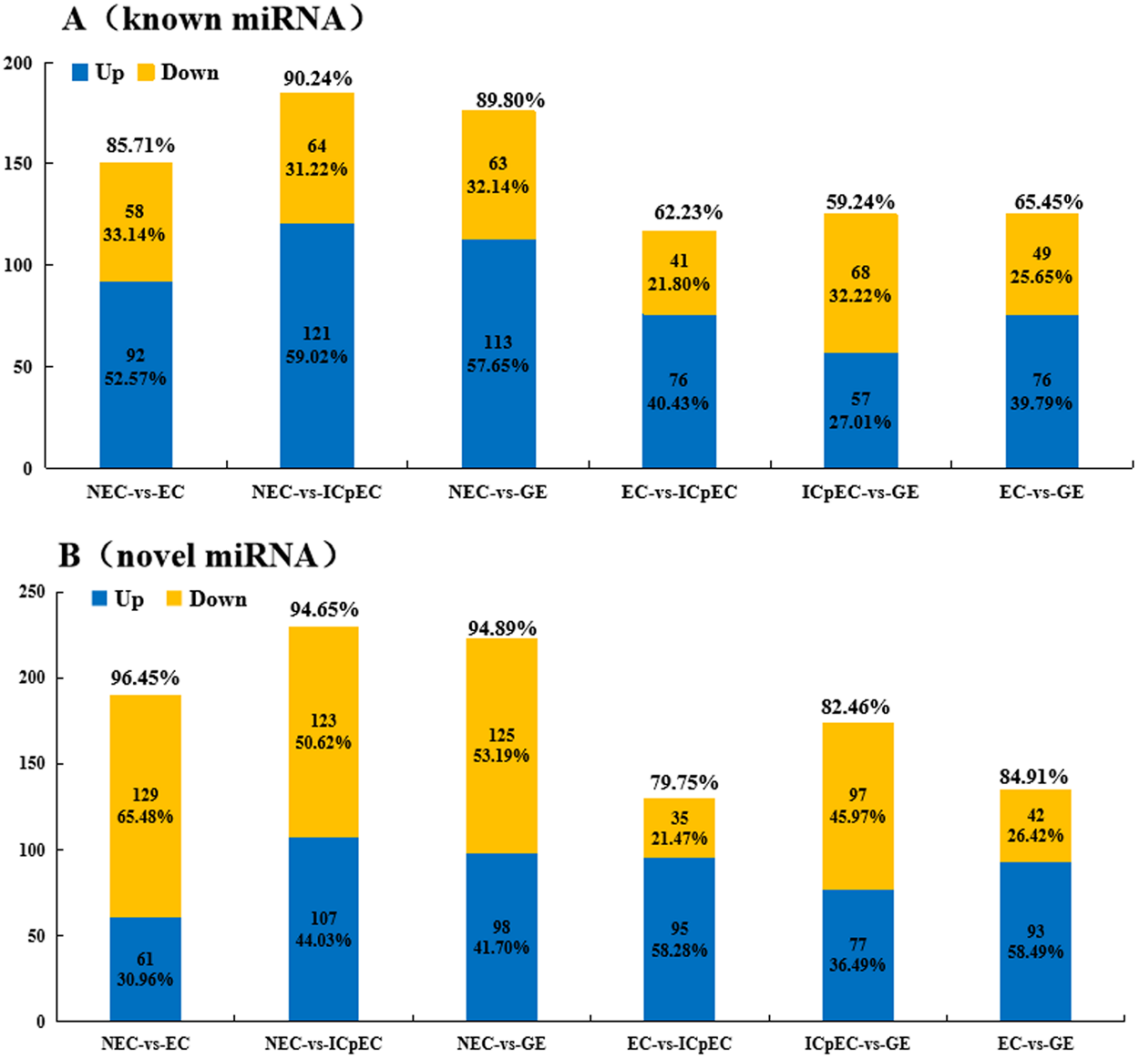
The number of known and novel up-regulation/down-regulation of miRNA in each group in the early SE and NEC.

A total of 289 known miRNAs and 1084 novel miRNA were obtained in the four stages (EC, ICpEC, GE, and NEC). In total, 150, 185, 176, 125, 117, and 125 known DE miRNAs (Fig. 3A), and 190, 230, 223, 130, 174, and 135 novel miRNAs (Fig. 3B) in NEC vs. EC, NEC vs. ICpEC, NEC vs. GE, EC vs. ICpEC, ICpEC vs. GE, and EC vs. GE, respectively.

**Figure 4:**
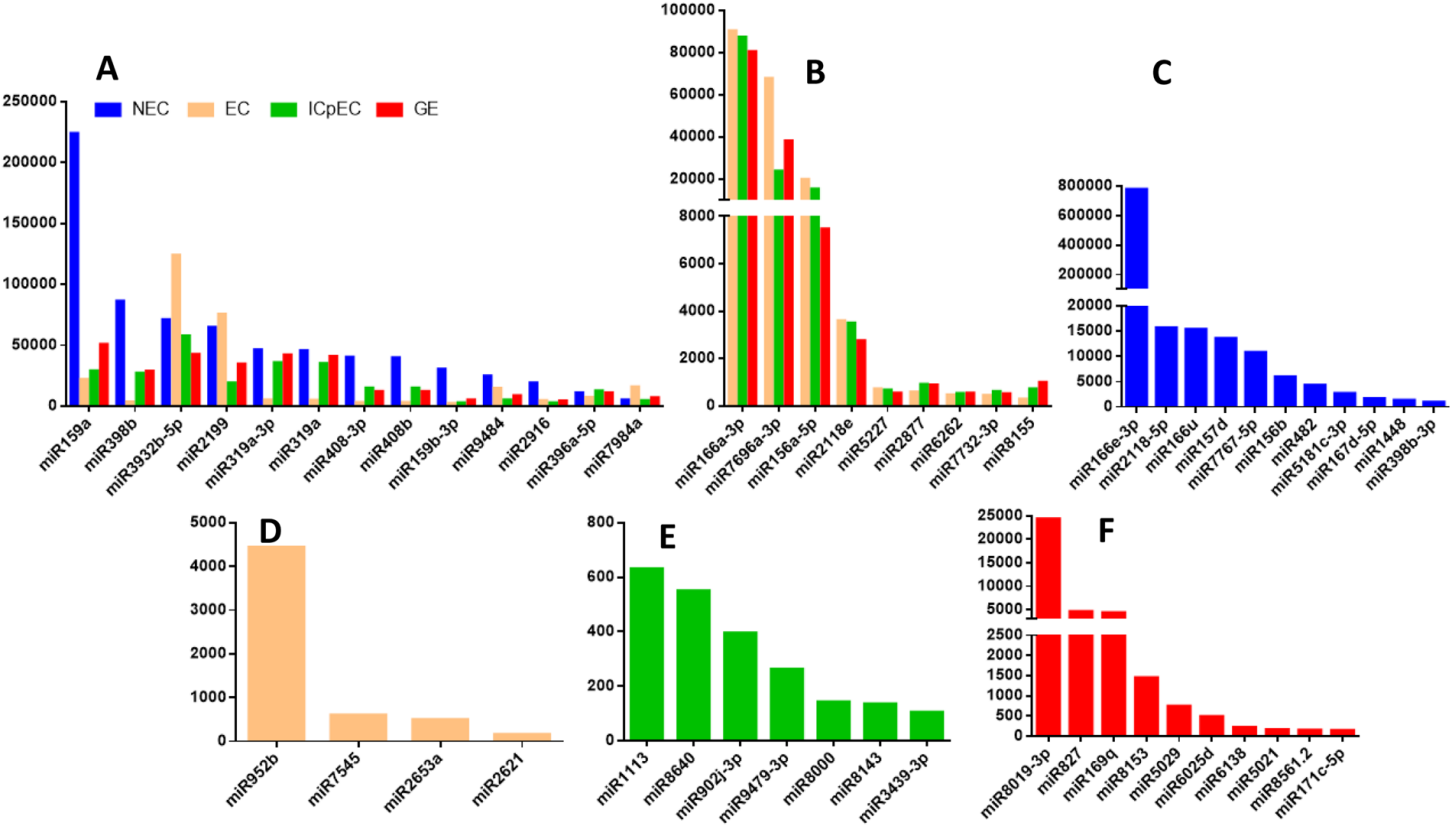
Differential expression pattern of miRNA in early SE and NEC of longan.

**Figure 5:**
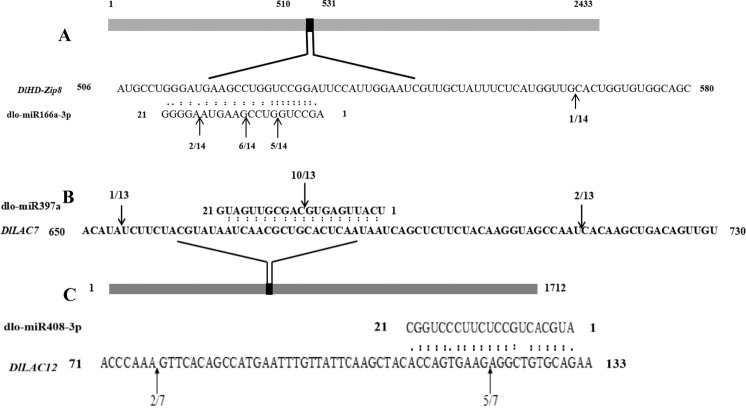
Identification of cleavage sites between dlo-miR166a-3p, dlo-miR397a and dlo-miR408-3p and their target genes inlongan. “(**A**) cleavage site mapping of miR166a-3p target gene. mRNA sequence of *DlHD-Zip8* is aligned with dlo-miR166a-3p. (**B**) cleavage site mapping of dlo-miR397a target gene. mRNA sequence of *DlLAC7* is aligned with dlo-miR397a. (**C**) cleavage site mapping of dlo-miR408-3p target gene. mRNA sequence of *DlLAC12* is aligned with dlo-miR408-3p. Numbers indicate the fraction of cloned PCR products terminating at different positions.

**Figure 6:**
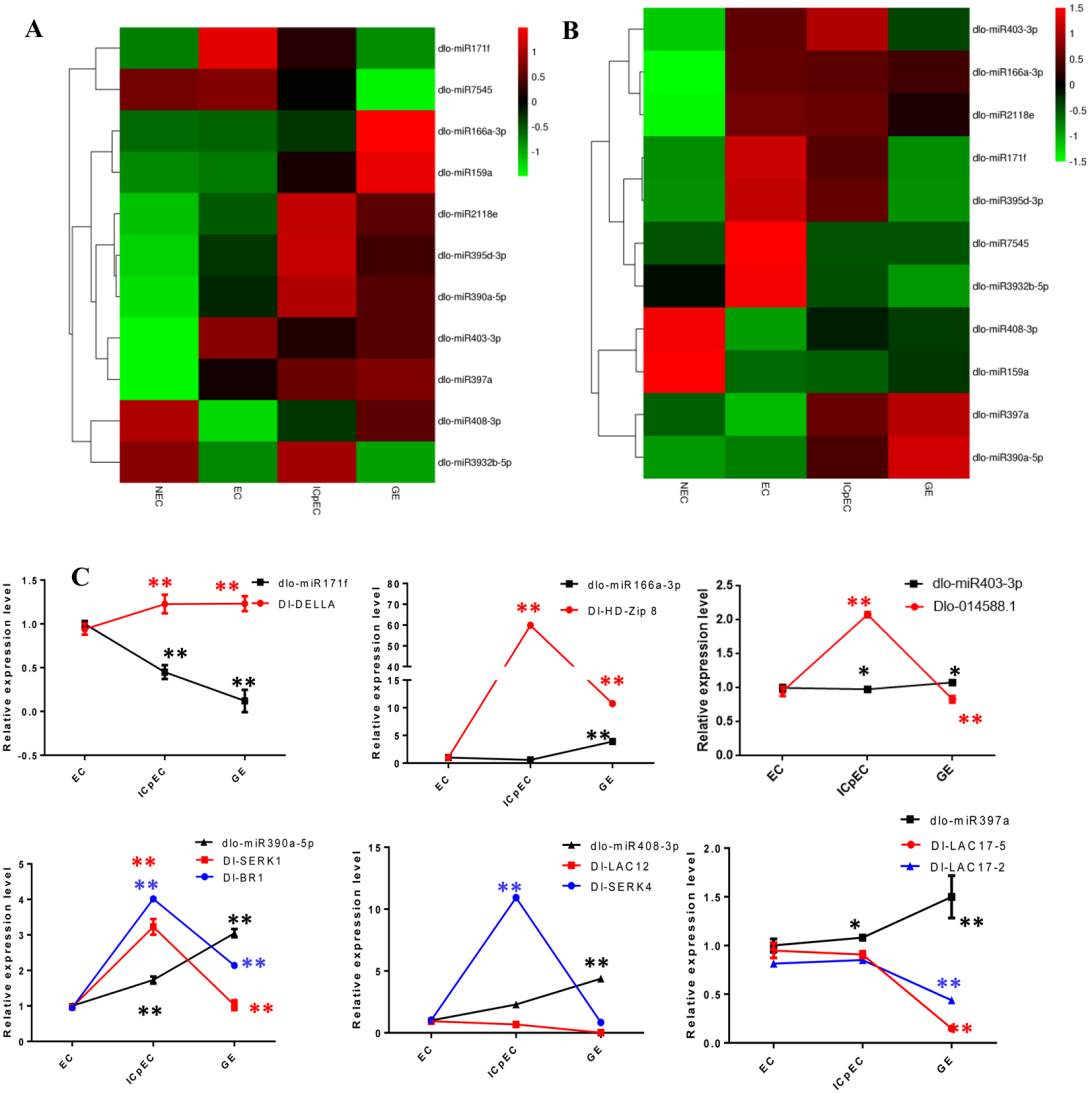
qRT-PCR expression patterns of 11 miRNAs in the early SE in longan. (**A**) relative expression levels in qRT-PCR. (**B**) is log2 (FPKM) values in the RNA-Seq library. The colors ranging from red to green indicate high to low correlation. (**C**) is the relative expressions of six miRNAs corresponding to the heat map and their potential target genes. *U6* snRNA was used as a reference gene to normalize miRNA and *DlUBQ* was used as a reference gene to normalize mRNA expression data. “ *” indicate significant difference at p-value < 0.05,“**” indicate significant difference at p-value < 0.01.

The fold-change values of DE miRNAs during the different stages were presented in Supplemental Data S9, Fig. 4c. According to the miRNA reads in the different stages, five different expression patterns were identified (Supplemental Data S10, Fig. 5). (i): Fifty-two miRNAs were expressed in four stages, and the read counts of 13 miRNAs (dlo-miR159a, dlo-miR398b, dlo-miR3932, dlo-miR319a-3p, dlo-miR408–3p, and dlo-miR319) were higher than 10,000 (Fig. 5A). The highest expressed miRNA in NEC was dlo-miR159a; dlo-miR3932b-5p and dlo-miR2199 were highly expressed in EC. (ii): Fourty miRNAs were expressed only in early SE, and the read counts of 10 miRNAs (dlo-miR166a-3p, dlo-miR7696a-3p, dlo-miR156a-5p, dlo-miR2118 among others) were higher than 500 (Fig. 5B). (iii): Forty-three miRNAs were expressed only in NEC, and the reads counts of 10 miRNAs were higher than 100 (dlo-miR166e-3p, dlo-miR2118-5p, dlo-miR166u, dlo-miR157d, dlo-miR482, and dlo-miR398b-3p among others.) (Fig. 5C). The highest expressed was dlo-miR8019-3p. (iv): Nineteen miRNAs were specifically expressed in EC, and the reads counts of four miRNAs (dlo-miR952b, dlo-miR7545, dlo-miR2653a, dlo-miR2621 among others.) were higher than 100 (Fig. 6D). (v): Forty-two miRNAs were expressed only in ICpEC, and seven miRNAs (dlo-miR1113, dlo-miR8640, dlo-miR902j-3p among others.) were higher than 100 reads, (Fig. 6E). (vi):Forty-one miRNAs were only expressed in GE, such as dlo-miR827, dlo-miR169q, dlo-miR171c-5p (Fig. 5F). There were great differences among different members of the same miRNA family in the expression of miRNA. For example, in dlo-miR166 family, the reads counts of different members varied from 0 to 777,715; dlo-miR166e-3p was only highly expressed in NEC. However, dlo-miR166a-3p was only expressed in early SE. Based on the above analysis, according to the fold change value, 11 miRNAs were selected and verified by qRT-PCR in early SE of longan.

In addition, novel DE miRNAs analysis showed that three novel miRNAs (novel_mir_207, novel_mir_34, and novel_mir_195) were characteristically up-regulated in EC vs. ICpEC, five novel miRNAs (novel_mir_38, novel_mir_100, novel_mir_170, novel_mir_60, and novel_mir_153) were specificly down-regulated in ICpEC vs. GE. Eleven novel_miRNAs (novel mir_309, novel mir_328, novel mir_361, novel_mir_359 among others.) were specifically up-regulated in EC vs. GE (Supplemental Data S11).

The above result showed that it was necessary to further analyse the DE miRNAs important functions of specifically expressed in the comparisons of NEC vs. EC, EC vs. ICpEC, ICpEC vs. GE and EC vs. GE.

A indicated that there was at least one miRNA reads more than 10000 (13) in the four stages; B mean that it was only expressed in all three stages of EC, ICpEC, GE, at least one miRNA reads more than 500. C indicated that 11 miRNA only in NEC stage, D indicated that only 4 miRNAs were expressed in the EC, E indicated that 7 miRNAs were expressed specifically in the ICpEC, F indicated that 10 miRNAs were expressed specifically in the GE, which reads all more than 100.

### Target prediction of DE miRNAs during early SE in longan

To analyse the biological functions of miRNAs in early SE and NEC, the target genes of miRNAs were predicted. The results showed that the known miRNAs of the four stages (EC, ICpEC, GE, NEC) regulated 1690, 1446, 1566 and 1888 target genes. Novel miRNAs targeted 1250, 2438, 2482 and 3543 candidate genes, respectively. Many potential target genes weren not TFs, such as LRR receptor-like serine/threonine protein kinases, DNA-directed RNA polymerases I II and III subunit RPABC1 (*POLR1C*), DELLA protein, ATP-dependent DNA helicase HFM1/MER3, (cytosine 38-c5)-methyltransferase, *MOB1*, acylaminoacyl-peptidase (*AAP*) and L-ascorbic acid oxidase (Supplemental Data S12). The TF-encoding genes, including *MYB*, zinc-binding motif transcription factors and auxin response factor (*ARF*).

As for the functional annotation of KO in the different stages. Among the non-TF targets, (cytosine 38-c5)-methyltransferase was annotated 50 times in NEC, but only 14–17 times in early SE; ascorbic acid oxygenase was annotated only 13 times and LRR receptor-like serine/threonine protein kinase annotated 31 times; notably, *AAP* was annotated 185 times in NEC, but only 10 times in early SE. *AAP* was targeted by dlo-miR8011a-5p and dlo-miR1044-3p. These miRNAs were up-regulated three-fold and eight-fold in SE, respectively. In NEC, miR5674a up-regulated 11-fold and targeted *AAP*, it might inhibit the development of early SE, but it was not discovered during embryogenesis for other plants. In contrast, the annotated frequency of *POLR1C* was the lowest in NEC (only 48 times). Meanwhile, in early SE, the frequency of *POLR1C* was annotated 60 times; dlo-miR9484 and dlo-miR2118a-3p mainly regulated *POLR1C*. The annotated frequency of *MOB1* was 15 times in EC, 12 times in NEC, but only 10 times in early SE; *MOB1* was mainly targeted by dlo-miR7545, dlo-miR394a, dlo-miR168a-3p, dlo-miR529, dlo-miR2118a-3p, dlo-miR7696a-3p, dlo-miR9484 among others. Compared with NEC, dlo-miR2118a-3p was down-regulated five-fold and dlo-miR7545 was up-regulated twelve-fold. Notably, some members of *MOB1* and *POLR1C* were concurrently regulated by dlo-miR2118a-3p and dlo-miR9484.

In addition, in order to further clarify the functions of DE miRNAs in different stages, dlo-miR166a-3p specifically targeted homeobox-leucine zipper protein (*DlHD-Zip)* in early SE. dlo-miR2118e mainly regulated two ATP-binding cassettes, subfamily G and phosphomannomutase. dlo-miR482a mainly regulated seven target genes, including *POLR1C* and Ca^2+^-transporting ATPase. dlo-miR408b regulated Cu^2+^ output ATP enzyme and *DlLAC12*, while dlo-miR397a targeted 12 including L-ascorbic acid oxidase and protein serine/threonine kinase. dlo-miR393h targeted *DlTIR1*. In EC vs. ICpEC, the main DE miRNA was dlo-miR390a-3p, which mainly regulated ribosomal biogenetic protein *DlBMS1*. In EC vs. GE, dlo-miR390a-5p was specifically up-regulated and targeted basic amino acid/polyamine antibody, APA family, brassinosteroid kinase inhibitor 1 (*BR1*), somatic embryogenesis receptor kinase 1 (*SERK1*), LRR receptor-like serine, HSP 20 family proteins and others. dlo-miR3932b-5p was specifically down-regulated in GE, which could target thirty-two genes such as centromeric protein and ESCRT-I complex subunit vps 28. dlo-miR4414 targeted WAT 1-associated protein and regulated cell secondary wall deposition. Moreover, dlo-miR395a targeted six ATP vulcanase 1 genes (Supplemental Data S13).

### GO and KEGG functional analyses of DE miRNAs

To further elucidate the potential pathways of the candidate target genes during early SE and NEC, GO and KEGG analyses were used to investigate the functions of putative target genes in early SE. GO enrichment analysis showed that the target genes of DE miRNAs were mainly enriched in three categories (Supplemental Data S14): biological process (BP), molecular function (MF) and cellular component (CC). In the four stages, the target genes were concentrated in ‘cellular process’, ‘metabolic process’, ‘catalytic activity’, ‘binding’, ‘cell’, ‘cell part’ and ‘single-organism process’. BP analysis showed that cytokinin-activated signalling pathway, lignin metabolic process, cell cycle process and DNA replication were significantly enriched in the comparison of NEC vs. EC (Supplemental Data S14). Lignin metabolism and DNA replication were significantly enriched in EC vs. ICpEC; however, lignin metabolism was only found in EC vs. GE. This suggested that the lignin metabolism pathway and cytokinin activated signalling pathway were particularly important in embryogenic induction (NEC vs. EC) of longan, and the lignin metabolism pathway plays a major role in early SE.

KEGG analysis was used to investigated target genes of the DE miRNAs in signal pathways; the threshold of FDR ≤ 0.05 was calculated using the corrected Q-value. Overall, 856, 743, 812 and 1121 target genes were annotated in EC, ICpEC, GE and NEC, respectively. The enrichment analysis showed that: in NEC vs. EC, 1129 target genes were enriched in 230 metabolic pathways. In addition, 597 target genes were enriched in 195 pathways in EC vs. ICpEC; 684 target genes were enriched in 208 pathways in EC vs. GE and 449 target genes were enriched in 170 pathways in ICpEC vs. GE.

The first 20 enrichment pathways of KEGG were summarised (Table 2). Some metabolic pathways were only enriched in one comparison. For example, sulfur metabolism, glucuronide biosynthesis, glucoside biosynthesis-ganglion cell series, trialkane, piperidine and pyridine alkaloid synthesis were only enriched in NEC vs. EC, these metabolic pathways might be closely related to embryonic totipotency. The metabolic pathway of glyoxylate and dicarboxylate metabolism was only identified in EC vs. GE. Some metabolic pathways were only found in two comparisons: in NEC vs. EC and NEC vs. ICpEC brassinosteroid biosynthesis, plant hormone signal transduction and collecting duct acid were mainly founded These pathways might be representative in the differences of physiological, biochemical and morphological between non-embryogenic and embryogenic stages. Tyrosine metabolism, benzoate degradation, aminobenzoate degradation and ethylbenzene degradation might involve in the comparisons of EC vs. GE and ICpEC vs. GE. The DE genes with basal cell carcinoma and circadian rhythm only occurred in the comparisons of EC vs. ICpEC and NEC vs. ICpEC. Some pathways appeared in three or five comparisons at the same time: for example, RNA degradation and ribosome biogenesis in eukaryotes occurred in NEC vs. EC, NEC vs. ICpEC and NEC vs. GE. Ascorbate metabolism and aldarate metabolism were mainly concentrated in the four groups of EC vs. GE, EC vs. ICpEC, NEC vs. EC and NEC vs. ICpEC. Apoptosis NF-kappa B signalling pathway and neurotrophin signalling pathway appeared in EC vs. GE, EC vs. ICpEC, ICpEC vs. GE and NEC vs. GE (Table 2), surprisingly, these two pathways in plants have been realy reported. However, plant pathogen interaction and pyrimidine metabolism appeared in six groups, suggesting that these two enriched pathways played important roles in early SE and NEC.

**Table 2:** The top 20 KEGG pathways enriched by target genes of DE miRNA in 6 groups.

pathway_term	group	pathway_term	group	pathway_term	group
Plant-pathogen interaction		Cytosolic DNA-sensing pathway		Ethylbenzene degradation	
Pyrimidine metabolism		RNA polymerase		Tyrosine metabolism	
Measles		Epste in-Barr virus infection		Benzoate degradation	
Ascorbate and aldarate metabolism		Huntington’s disease		Limonene and pinene degradation	
NF-kappa B signaling pathway		Purine metabolism		Aminobenzoate degradation	
Neurotrophin signaling pathway		Ribosome biogenesis in eukaryotes		Plant hormone signal transduction	
Apoptosis		RNA degradation		Collecting duct acid	
Toll-like receptor signaling pathway		Basal cell carcinoma		Homologous recombination	
Leishmaniasis		Circadian rhythm - fly		Brassinosteroid biosynthesis	
Toxoplasmosis		htlv-i infection		Legionellosis	
Pertussis		p53 signaling pathway		Ether lipid metabolism	
Influenza A		Ubiquitin mediated proteolysis		Sulfur metabolism	
Chagas disease (American trypanosomiasis)		Transcriptional misregulation in cancer		Tropane,piperidine and pyridine alkaloid biosynthesis	
Glyoxylate and dicarboxylate metabolism		Antigen processing and presentation		Glycosphingolipid biosynthesis - ganglio series	
Tuberculosis		Spliceosome		Glucosinolate biosynthesis	
Fanconi anemia pathway		Taurine and hypotaurine metabolism			

Notes: : EC-vs-GE; :EC-vs-ICpEC; : ICpEC-vs-GE; :NEC-vs-EC; : NEC-vs-GE; : NEC-vs-ICpEC.

#### Lignin metabolism pathway during early SE in longan

GO enrichment analysis of the target genes showed that lignin metabolism (GO: 0009808) appeared in four stages (EC, ICpEC, GE, and NEC). Lignin metabolism and cytokinin metabolism were mainly identified in the comparison of NEC vs. EC. Moreover, dlo-miR397a targeted 12 laccase genes (*Dlo_023717.1*, *DlLAC17-5*; *Dlo_031945.1*, *DlLAC4-3*; *Dlo_011454.1*, *DlLAC19*; *Dlo_007669.1*, *DlLAC17-1*; *Dlo_011079.1*, *DlLAC11-2*; *Dlo_024904.2*, *DlLAC7*; *Dlo_029429.2*, *DlLAC4-2*; *Dlo_023718.1*, *DlLAC18-2*; *Dlo_000026.1*, *DlLAC11-1*; *Dlo_007670.1*, *DlLAC17-2*; *Dlo_023721.1*, *DlLAC17-7*; and *Dlo_007673.1*, *DlLAC17-3*) to regulate lignin metabolism. *Laccase* (*LAC*) was not only involved in the metabolism of phenol-related substances but also participates in the lignin metabolism pathway in early SE. The expression of dlo-miR397a was up-regulated in early SE compared with NEC, indicating that the accumulation of dlo-miR397a might promote the development of embryogenesis and early SE in longan. It was inferred that the dynamic changes of lignin metabolism might affect the SE development of longan.

#### Sulfur metabolism pathway of embryogenesis in longan

According to the analysis of enrichment factors in the KEGG metabolic pathway. Sulfur metabolism and glucosinyl metabolism were only identified in the comparison of NEC vs. EC, which were regulated by four miRNAs (dlo-miR2592ae, dlo-miR7539, dlo-miR7545, and dlo-miR952b). dlo-miR2592ae regulated two desulfoglucosinolate sulfotransferases a/b/c (dsGSs), while dlo-miR7539 regulated two sulfite oxidases. dlo-miR7545 targeted a thioglucose-thiotransferase a/b/c. dlo-miR952b targeted 3H-adenosine phosphate 5-phosphate sulfate synthase (*PAPS*). However, sulfur metabolism and glucosinyl metabolism were not to be activated in other stages, this pathway might play a leading role in NEC and EC (Supplemental Data S15, Fig. 4b).

#### Alternative splicing and ubiquitin-mediated proteolytic pathways in EC and ICpEC

According to the first 20 KEGG metabolic pathways in longan, the alternative splicing pathway only occurred in the comparison of EC vs. ICpEC. dlo-miR162a-5p regulated splicing factor 3B subunit 4, while dlo-miR2118e regulated two members of the ABC transporter G family. dlo-miR3512 regulated pre-mRNA-splicing helicase BRR2. dlo-miR5083 regulated U4/U6 small nuclear ribonucleoprotein PRP4. dlo-miR5540 regulated heat shock 70 kDa protein. dlo-miR7545 targeted splicing factor 3B subunit 3, as well as ATP-binding cassette, subfamily B (MDR/TAP), and PRP22. dlo-miR952b also targeted polyglutamine-binding protein 1 and THO complex subunit 2. Among others, dlo-miR5183, dlo-miR5083, dlo-miR7545 and dlo-miR952b were down-regulated 7–15 times, which suggested that these miRNAs might be the main participants in the development of ICpEC. These miRNAs targeted splicing dexd/h cassette ATP enzymes such as prp3, prp4, prp8, prp16 and prp22, which constituted the protein shear complex. Therefore, it was inferred that the development of ICpEC not only involved biological processes of cell division, proliferation and DNA replication, as well as alternative splicing events.

In addition, the ubiquitin-mediated proteolysis pathway was only identified in the comparison of EC vs. ICpEC. dlo-miR319a and dlo-miR319a-3p targeted *STIP1* and E3 ubiquitin protein ligase hc4, dlo-miR398a-3p regulated E3 ubiquitin protein ligase TRIP 12, dlo-miR2873b targeted *SERK1*. E3 ubiquitin protein ligase HERC4 was regulated by three miRNAs (dlo-miR319a, dlo-miR319a-3p, and dlo-miR398a-3p) (Supplemental Data S15, Fig. 4b).

#### Plant hormone signal transduction pathways in early SE

Based on the analysis of KEGG enrichment pathways, the DE miRNAs regulated target genes that participate in plant hormone signal transduction pathways, including auxin metabolism and brassinolactone metabolism. dlo-miR2631 regulated *AUX1* and dlo-miR393h regulated *TIR1* in the auxin signalling pathway in the embryogenic development of longan. dlo-miR5181c-3p/dlo-miR2592ae/dlo-miR5181c-3p/dlo-miR157a-3 targeted four *AUX/IAA* genes and dlo-miR2592ae targeted three *ARF* genes. These miRNAs were down-regulated eignt–fourteen-fold in EC and ICpEC. These results supported that the auxin pathway acts as a switch to regulate the embryogenic ability of longan callus. dlo-miR2631, dlo-miR393h, dlo-miR5181c-3p/dlo-miR2592ae/dlo-miR5181c-3p/dlo-miR157a-3, dlo-miR2592ae and dlo-miR827-5p played important roles in auxin metabolism of early SE in longan.

The brassinosteroid biosynthesis pathway was specifically enriched in the comparisons of NEC vs. EC and NEC vs. ICpEC, and was one of the top 20 KEGG enriched pathways. Four isocitrate dehydrogenase genes (dlo_036406.1, dlo_034642.1, dlo_038278.1, and dlo_038654.1) were targeted by dlo-miR5181c-3p, which might be involved in the synthesis of 22-hydroxycampesterol during steroid biosynthesis. In the comparison of NEC vs. EC, dlo-miR5054 regulated rapese-6-oxidase 2 (Dlo_004309.2), which catalyses BL brassolactone to synthesise typha sterol (TY), theosterone (TE), brassinone (CS), and brassinolide (BRs); dlo-miR7696a-3p, dlo-miR6221-3p, and dlo-miR5083/miR6300 regulated three *PHYB1* genes (Dlo_019439.1, Dlo_020565.1, and Dlo_020585.1), and involved in regulating the synthesis of 26-hydroxybrassinosterone (hydroxycastasterone) and 26-hydroxybrassinolide (hydroxybrassinolide). This process was uniquely identified in the comparisons of NEC vs. EC and NEC vs. ICpEC. Some miRNAs targeting the three *PHYB1* genes were up-regulated three–seventeen-fold, which suggested that the lower accumulation of brassinolide might promote early embryogenesis development of longan callus (Supplemental Data S15).

### Relationships between dlo-miR166a-3p, dlo-miR397a, dlo-miR408-3p, and their targets were confirmed by modified RLM-RACE

According to the DE miRNA which might paticipate the lignin metabolism pathway of longan, the relationships between dlo-miR166a-3p, dlo-miR397a, dlo-miR408-3p and their target genes were confirmed by RLM-RACE in early SE (Fig. 6). The results showed that dlo-miR166a-3p could regulate *DlHD-zip8*, three cleavage sites were located at the 6th CUG/GUC, the 10th site was UCC/GAA, and the 16th was GUA/AGG of the dlo-miR166a-3p. Moreover, the highest probability of cleavage sites occurred at the 10th site. A cleavage site might be located outside of the targeted complementary region, but the probability of this was small. dlo-miR397a targeted *DlLAC7* with three cleavage sites, one of the most typical cleavage sites was located at the 10th site GUG/CAG. The other two cleavage sites were located outside of the complementary region. dlo-miR408-3p targeted *DlLAC12* with two cleavage sites; the most typical cleavage site was the 10th site AAG/AGG. The other cleavage site was located outside of the complementary area. In conclusion, dlo-miR166a-3p, dlo-miR397a and dlo-miR408-3p tended to cleave their target genes at the 10th site, but they might also target sites outside of the target complementary region and be targeted by other miRNAs.

### qRT-PCR analysis of miRNAs and their target genes during early SE in longan

**Figure 7:**
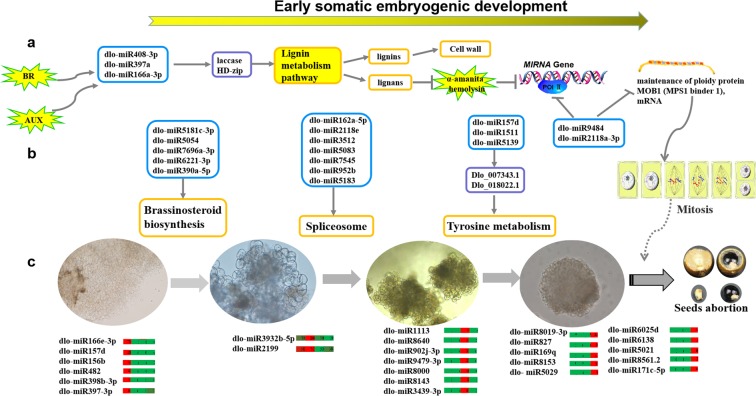
Possible regulation network draft of miRNA in the early SE of longan. a represent some miRNA may paticipate in the lignin pathway and regulate RNA polymerase II. b represent some miRNA may paticipate in Brassinosteroid biosynthesis, Spliceosome, Tyrosine metabolism regulate the development of early SE. c represent some DE miRNA paticipate in different early SE of longan. (Note: This image was drawn by the author, according to the summary of discussion.).

The qRT-PCR expression patterns of 11 miRNAs were analysed in early SE and NEC. The expression patterns of eight miRNAs (dlo-miR166a-3p, dlo-miR7545, dlo-miR397a, dlo-miR390a-5p, dlo-miR2118e, dlo-miR171f, dlo-miR395d-3p, and dlo-miR408-3p) were consistent with the results of transcriptome sequencing, and divided into three expression patterns: (i) up-regulated expression (dlo-miR166a-3p and dlo-miR397a) in the four stages; (ii) down-regulated expression (dlo-miR7545 and dlo-miR408-3p) in the early SE; and (iii) ‘inverted V-shaped’ expression pattern (dlo-miR390a-5p, dlo-miR2118e, dlo-miR171f, dlo-miR408-3p, dlo-miR395d-3p) in early SE and NEC. The expression pattern of three miRNAs (dlo-miR403-3p, dlo-miR3932b-5p, dlo-miR159a) were partially consistent with the results of RNA-sequencing (Fig. 7).

The three miRNAs (dlo-miR390a-5p, dlo-miR408-3p, and dlo-miR397a) up-regulated in the early SE, the target gene *DlBR1* and *DlSERK1* of dlo-miR390a-5p down-regulated in GE. *DlSERK4* was down-regulated and targeted by dlo-miR408-3p in GE. *DlLAC17-2* and *DlLAC17-5* were down-regulated in GE and targeted by dlo-miR397a. The results showed that *DlBR1*, *DlSERK1*, *DlSERK4*, *DlLAC17-2* and *DlLAC17-5* were negatively regulated by dlo-miR390a-5p, dlo-miR408-3p and dlo-miR397a at ICpEC and GE. *DlLAC12* was another target gene of dlo-miR408-3p, and down-regulated in the early SE. The expression levels of dlo-miR166a-3p and dlo-miR403-3p decreased slowly and then increased in the early SE, while the expression levels of their target genes, *DlHD-zip 8* and *Dlo-014588.1*, first increased and then decreased. However, the expression level of dlo-miR171f decreased gradually in early SE of longan, and its target gene, *DlDELLA*, was up-regulated. Therefore, in longan early SE, most of the miRNA expression patterns were consistent with the sequencing results, and the negative regulatory relationships between target genes and miRNAs were verified, this further indicated that the RNA-seq results were credible (Fig. 7).

## Discussion

### Many miRNAs are involved in early SE in longan

A previous study demonstrated that miRNAs participate throughout the development of somatic embryogenesis in longan^11^, but did not includ a large-scale genome-wide analysis of the expression levels of miRNAs in every stage of somatic embryogenesis in longan. In our study, more than 100 conserved miRNAs were measured in every stage. Among them, dlo-miR159a, dlo-miR398b, dlo-miR3932, dlo-miR319a-3p, dlo-miR408-3p and dlo-miR319 were expressed throughout the development of SE and NEC. Our findings generally support those reported by Lin^11^, it was beneficial for the development of embryogenic callus to down-regulate dlo-miR159a and dlo-miR319. Moreover, in our study, the accumulation of dlo-miR159 (dlo-miR159a and dlo-miR159b-3p) was beneficial for the development of GE. In *Arabidopsis*, miR159 was shown to regulate *MYB33* and was induced by ABA during seed germination^41^, while the accumulation of ABA was increased from NEC to EC^27^. This is consistent with the participation of miR159 in ABA signal transduction and its promotion of embryonic induction, similar to the findings of our study. dlo-miR398b was found to be highly expressed in heart embryo, torpedo embryo and cotyledonary embryos during sequencing of sample mixtures from nine stages^11^. In our study, dlo-miR398b was higher expressed in NEC than in early SE, and particularly highly expressed in GE. Besides, we found that a high expression level of dlo-miR408-3p was beneficial to NEC morphogenesis, while it was also up-regulated from EC to GE (Fig. 4a). In maize somatic embryogenesis, miR408, and miR398, as well as their target genes (*LAC2* and *SOD9*) exhibited substantial changes during the photoperiod exposure^42^, our study made up for previous research.

dlo-miR166a-3p, dlo-miR156a-5p, dlo-miR2118 and dlo-miR7696a-3p were stably highly expressed in early SE. dlo-miR166a-3p targeted *DlHD-zip III* (Fig. 7), which regulated GE morphogenesis; this further confirmed the findings of the research by Lin^11^. In *Arabidopsis*, miR166a might be induced by GA_3_ and regulate the auxin response^43^. We found that, although miR2118 and miR482 might belong to the same family present in many plants^44,45^, dlo-miR2118 was highly expressed in early SE, while dlo-miR482 was only highly expressed in NEC (4281 reads). miR482 exhibited sequence diversity and has been widely found in dicotyledonous plants, its target gene, NBS-LRR, could have evolved over time to respond to biological stress^44^. The expression pattern of dlo-miR482 was similar to that previously reported in *Citrus*^46^. In mulberry phloem sap, it was shown that miR482 could participate in two-way transportation during long-distance conduction^47^. In particular, in our study, dlo-miR8019-3p, dlo-miR171c-5p, dlo-miR827, dlo-miR169q, and dlo-miR8153 were specifically expressed in GE, which contrasted with the findings in a study by Lin^11^. The miR169 family is a large and conserved miRNA family in many plant species. In *Arabidopsis*, miR169 targeted the NF-YA3 transcription factor and was shown to be highly expressed in late SE induction (10 days)^48^; in addition, miR169 participats in nitrogen-starvation responses and stress^48,49^. dlo-miR169q was only highly expressed in GE of longan, which might be related to nitrogen or stress. In *Lilium* SE, miR169 was differentially expressed in NEC and EC1^17^. dlo-miR171c-5p was only expressed in GE, whereas dlo-miR171f was highly expressed in EC and ICpEC (Fig. 7), notablly, miR171c was largely accumulated in EC of *Citrus*^46^. miR827 could target NLA (Nitrogen Limitation and Adaptation) and PHT5 (Phosphate Transporter 5)^50^. Although it was conserved, dlo-miR827 targeted two glutathione S-transferases (Dlo_032407.1 and Dlo_032413.1) in longan. dlo-miR166e-3p, dlo-miR157d, dlo-miR156b, dlo-miR482, dlo-miR2118-5p, dlo-miR398b-3p and dlo-miR397-3p were not expressed in early SE, but only in NEC; inhibition of these miRNAs might promote the initiation of EC.

### dlo-miR397a, dlo-miR408-3p and dlo-miR166a-3p might participate in the development of early SE *via* the lignin metabolism pathway

miR397, miR408 and miR166 are conserved miRNAs in plants, which have been reported to participate in lignin and xylem synthesis^51–53^. *Laccase* and *HD-Zip* have also been widely studied^54–56^. However, there has been limited research regarding the associations between miRNAs and lignin regulation in plant embryo development, because target genes vary among species; moreover, miRNA biological functions are diverse and exhibit functional redundancy.

In our study, the expression levels of dlo-miR397a, dlo-miR408-3p, and dlo-miR166a-3p were up-regulated in early SE, especially in GE. Their target genes (*DlLAC17-2*, *DlLAC17-5*, *DlLAC12*, *DlSERK4*,and *DlHD-zip 8*) were down-regulated in GE, which might be related to the development of SE.

Lignin is one of the main components in the plant secondary cell wall, and is related to plant growth and differentiation^57^. In rice, OsmiR397 was strongly specifically expressed in undifferentiated embryogenic calli^58^, down-regulated *laccase* to vital to maintaining embryonic cells in a thin-wall and meristematic state. In *Citrus*, miR397 was highly expressed in globular embryos^10^, consistent with our results. In maize SE, the expression of miR408 and miR397 occurred in response to different concentrations of hormone in embryogenic tissues^42^. Our study suggested that dlo-miR408-3p might target *DlLAC12* and *DlSERK4*, but this hypothesis is not supported by the results of degradome sequencing^11^.

miR166/165 is an ancient and conserved miRNA family in plants. In *Arabidopsis thaliana*, miR166/165 can maintain the differentiation into stem apical meristem by influencing with AGO10, such that it cannot integrate into the AGO1 complex; this inhibits the expression of *HD-Zip III* and affects xylem formation and differentiation^59^. Our study found that the expression of dlo-miR166a-3p was significantly up-regulated in early SE, and most highly expressed in GE. Its target gene was *DlHD-Zip 8* (*ATHB8*), it was significantly more highly expressed in GE than that in NEC and EC. Zhiqian Li investigated the *HD-zip* family from the whole-genome analysis of ovules and somatic embryos in grape, and found that *HD-zip* genes family might regulate seed abortion^60^. However, *ATHB8* of the *HD-Zip* family was induced by auxin in *Arabidopsis thaliana*, and it was strongly expressed in vascular tissue when embryos developed to the heart embryonic stage^43^. In contrast, the accumulation of *DlHD-Zip 8* (*ATHB8*) occurred earlier in the early SE of longan than in *Arabidopsis thaliana* SE, which suggested that more complete vascular tissue might form in the longan GE. In *Arabidopsis* root, miR166 can cleave the mRNA of *HD-Zip III* to varying degrees, by moving from cortex to middle column in different gradient concentrations, it was shown to regulate the formation of epidermal xylem and primary xylem^61^. Similarly, the interaction of dlo-miR166 and *HD-Zip III* might involve in the regulation of xylem in longan early SE. In recent years, it has been shown that *HD-Zip III* might be induced by BR and be involved in the division and differentiation of procambium cells to produce xylem^62^. In this experiment, the auxin signalling pathway and brassinolide pathway were significantly enriched in the comparison of NEC vs. EC; dlo-miR166a-3p was not expressed in NEC, suggesting that it might play a major role in totipotency. The dlo-miR166a-3p might be induced by auxin and brassinolide. Hence, the changes in xylem cells and lignin metabolism might be regulated by dlo-miR166a-3p, dlo-miR408-3p, and dlo-miR397a, which might be affected by auxin and BR during the development of longan SE (Fig. 4a).

### The miRNAs targeted RNA polymerase II might play an important role in early SE of longan

RNA polymerase II not only involved in transcribing mRNA, but also played an important role in the process of miRNA biosynthesis; specifically, it transcribed miRNA genes into primary miRNAs (pri-miRNAs)^63^. Intriguingly, AAP was annotated more frequently in NEC than in early SE, it was targeted by dlo-miR8011a-5p and dlo-miR5764a, and it encoded proline oligopeptidase (POP) in plants. It was found that flavonoids and lignans could inhibit the activity of POP in *Rhodiola tangut*^64^. POP is the key enzyme in the synthesis of amanitatoxin, an α-ointment peptide. At low concentration, amanitatoxin could inhibit the activity of RNA polymerase II, there by hindering protein synthesis^65^. α-Ointment peptide was reported to inhibit the hypocotyl growth of mung bean seeds^66^. However, the annotated frequency of *POLR1C* and *MOB1* were only lowest abundance in NEC, highest abundance in EC and lower in ICpEC and GE. Both *POLR1C* and *MOB1* were regulated by the same miRNA (dlo-miR2118a-3p, dlo-miR9484), which was contrary to the abundance of *AAP* in the early SE*. MOB1* is a cell-ploidy maintenance protein and plays an important role in cell mitosis^67^, involved in the initiation of cell division, the coordination of cell polarity and the cell cycle during mitosis^68^. In *Arabidopsis thaliana*, down-regulation of the mob1 gene may cause cell cycle disorder and affect sporophyte and gametophyte development^69^, and may be involved in the regulation of growth and development via auxin signal transduction^70^. It may also involved in Hippo metabolic pathways, regulating cell division, withdrawal and polarity establishment to control tissue growth and programmed cell death, and determine the size of organs^71^. The most important of these processes must be regulated by binding Pol II. Therefore, we speculated that POP activity was higher in NEC, which might inhibit the activities of *POLR1C*, and hindered the synthesis of some proteins in NEC. This further supported the findings of Chen Chunling’s research, in which protein staining was very weak and protein metabolism was very low at NEC^27^. At the same time, *POLR1C* might also regulate *MOB1* to participate in cell mitosis controlling the size of organs^72–74^. Therefore, AAP (POP) might influence the biosynthesis of miRNA and cell mitosis by affecting the activity of Pol II and *MOB1* during the development of early SE in longan (Fig. 4a).

## Supplementary information

Supplementary Data.

Supplementary Data2.

Supplementary Data3.

Supplementary Data4.

Supplementary Data5.

Supplementary Data6.

Supplementary Data7.

Supplementary Data8.

Supplementary Data9.

Supplementary Data10.

Supplementary Data11.

Supplementary Data12.

Supplementary Data13.

Supplementary Data14.

Supplementary Data15.

## Data Availability

All the data generated or analyzed during this study are included in this published article and its Supplementary Information Files.
