# Supplementary material for: Genome-wide identification of miRNAs and their targets during early somatic embryogenesis in Dimocarpus longan Lour
Source: Sci Rep. 2020 Mar 13;10:4626. doi: 10.1038/s41598-020-60946-y (PMC7069941; doi:10.1038/s41598-020-60946-y)
Supplement: Supplementary file 14 — Supplementary Data14. [file 41598_2020_60946_MOESM14_ESM.pdf]

A

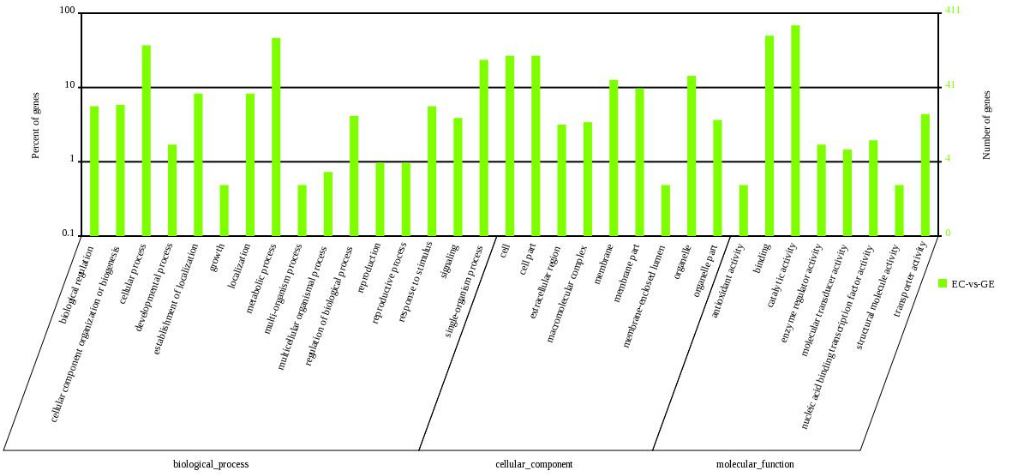

100
10
1
0.1
411
41
4
0
Percent of genes
Number of genes
biological regulation
cellular component organization or biogenesis
cellular process
developmental process
establishment of localization
growth
localization
metabolic process
multi-organism process
multicellular organismal process
regulation of biological process
reproduction
reproductive process
response to stimulus
signaling
single-organism process
cell
cell part
extracellular region
macromolecular complex
membrane
membrane part
membrane-enclosed lumen
organelle
organelle part
antioxidant activity
binding
catalytic activity
enzyme regulator activity
molecular transducer activity
nucleic acid binding transcription factor activity
structural molecule activity
transporter activity
EC-vs-GE
biological_process
cellular_component
molecular_function

B

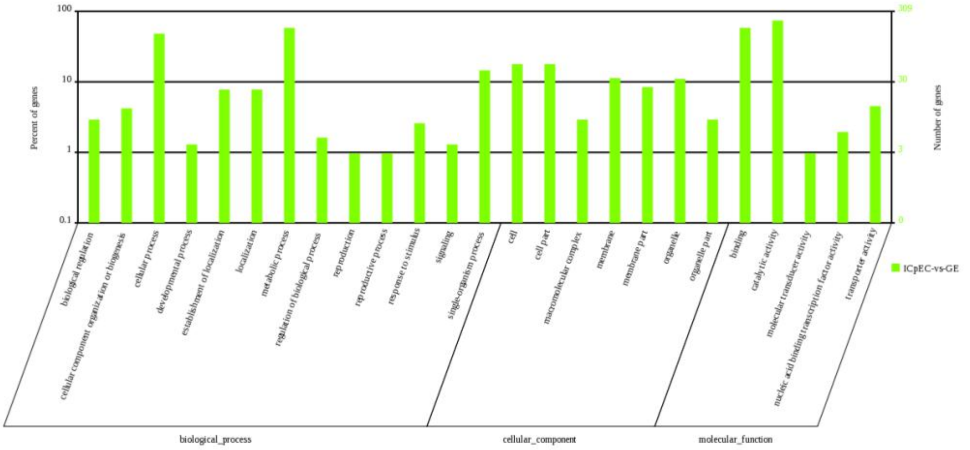

100
10
1
0.1
309
30
3
0
Percent of genes
Number of genes
biological regulation
cellular component organization or biogenesis
cellular process
developmental process
establishment of localization
localization
metabolic process
regulation of biological process
reproduction
reproductive process
response to stimulus
signaling
single-organism process
cell
cell part
macromolecular complex
membrane
membrane part
organelle
organelle part
binding
catalytic activity
molecular transducer activity
nucleic acid binding transcription factor activity
transporter activity
ICpEC-vs-GE
biological_process
cellular_component
molecular_function

C

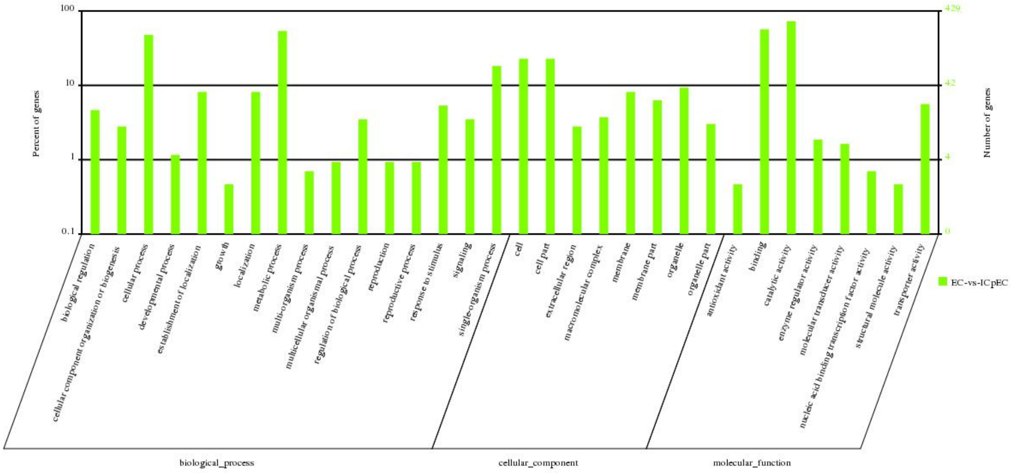

100
10
1
0.1
429
42
4
0
Percent of genes
Number of genes
biological regulation
cellular component organization or biogenesis
cellular process
developmental process
establishment of localization
growth
localization
metabolic process
multi-organism process
multicellular organismal process
regulation of biological process
reproduction
reproductive process
response to stimulus
signaling
single-organism process
cell
cell part
extracellular region
macromolecular complex
membrane
membrane part
organelle
organelle part
antioxidant activity
binding
catalytic activity
enzyme regulator activity
molecular transducer activity
nucleic acid binding transcription factor activity
structural molecule activity
transporter activity
EC-vs-ICpEC
biological_process
cellular_component
molecular_function

D

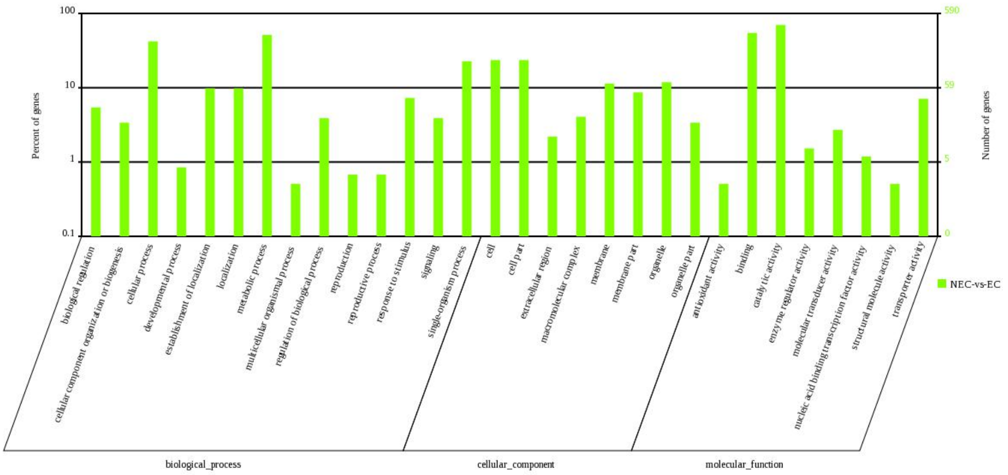

E

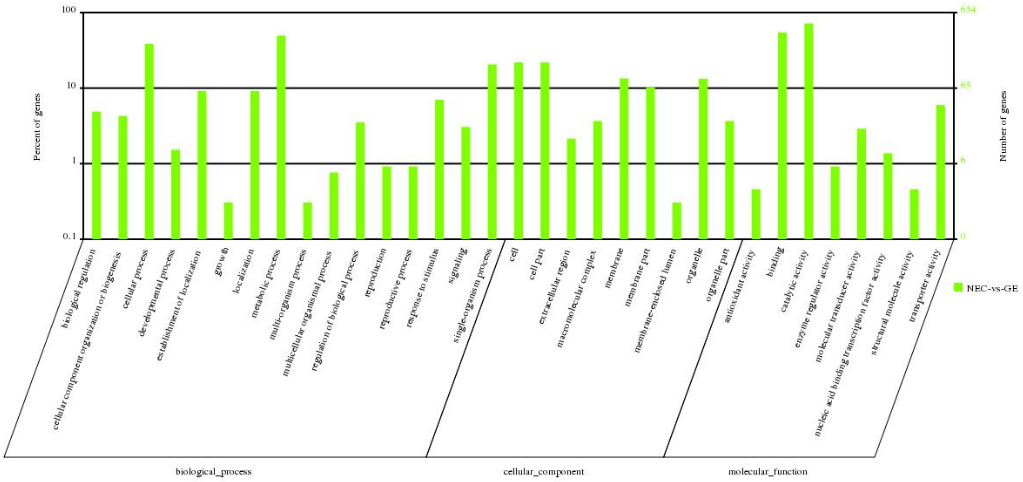

F

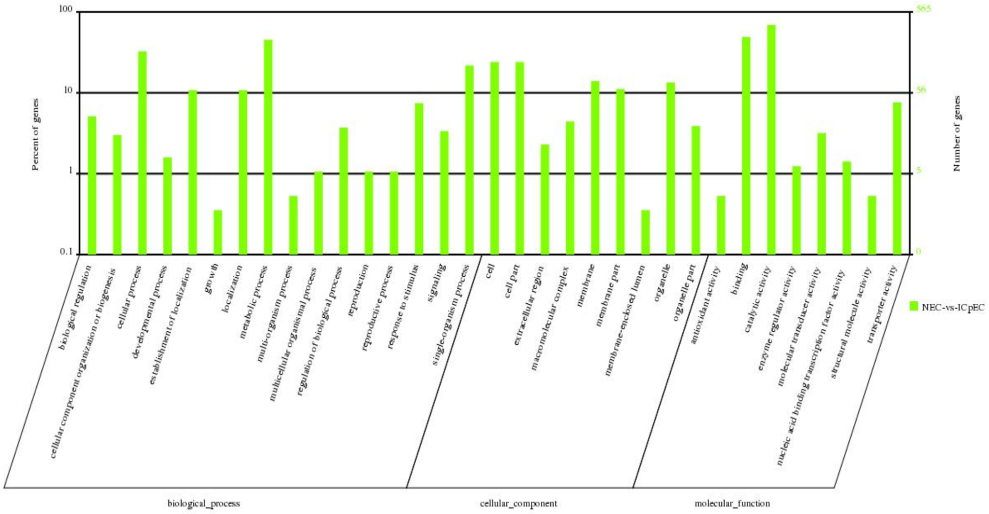

Figure S1. Enrichment analysis mapped to longan genome during different early somatic embryogenesis of longan.

A, B, C, D, E, F represent EC-*vs*-GE, ICpEC-*vs*-GE, EC-*vs*-ICpGE, NEC-*vs*-EC,NEC-*vs*-GE, NEC-*vs*-ICpGE.
