# Supplementary material for: Genome-wide identification of miRNAs and their targets during early somatic embryogenesis in Dimocarpus longan Lour
Source: Sci Rep. 2020 Mar 13;10:4626. doi: 10.1038/s41598-020-60946-y (PMC7069941; doi:10.1038/s41598-020-60946-y)
Supplement: Supplementary file 15 — Supplementary Data15. [file 41598_2020_60946_MOESM15_ESM.pdf]

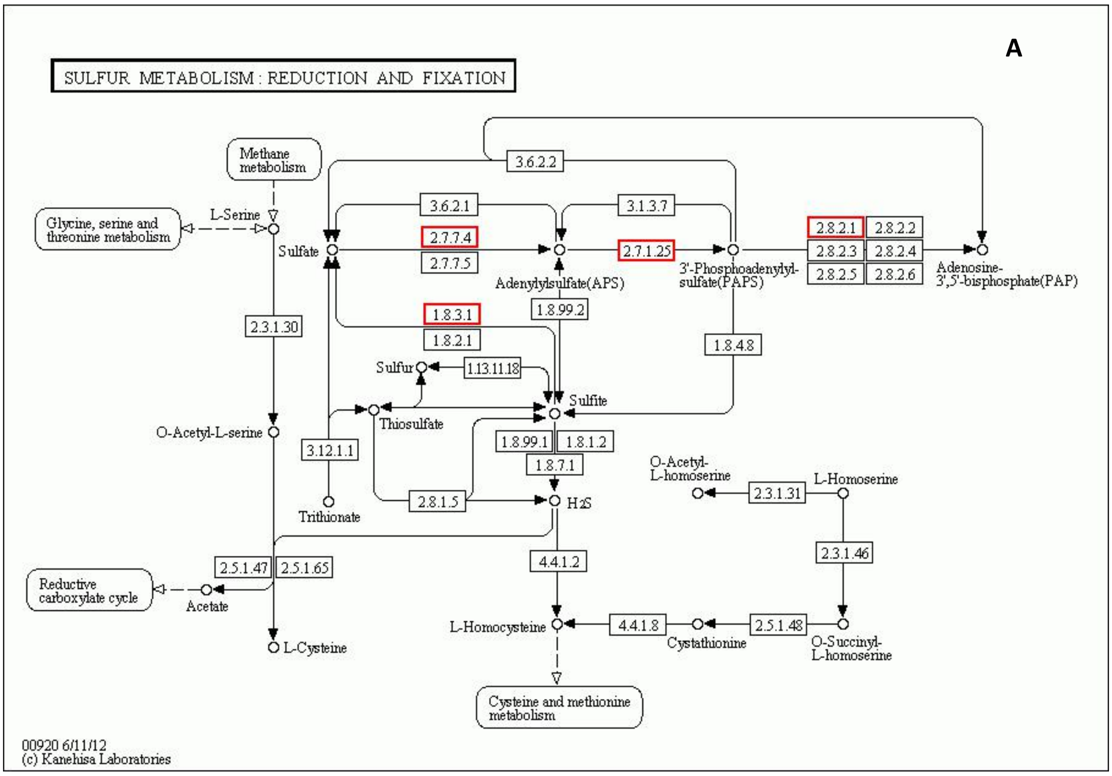
A
SULFUR METABOLISM : REDUCTION AND FIXATION
Methane metabolism
Glycine, serine and threonine metabolism
L-Serine
Sulfate
3.6.2.2
3.6.2.1
3.1.3.7
2.7.7.4
2.7.7.5
2.7.1.25
Adenylylsulfate(APS)
3'-Phosphoadenylyl-sulfate(PAPS)
2.8.2.1
2.8.2.2
2.8.2.3
2.8.2.4
2.8.2.5
2.8.2.6
Adenosine-3',5'-bisphosphate(PAP)
2.3.1.30
1.8.3.1
1.8.2.1
1.8.99.2
1.8.4.8
Sulfur
1.13.11.18
Sulfite
O-Acetyl-L-serine
3.12.1.1
Thiosulfate
1.8.99.1
1.8.1.2
1.8.7.1
O-Acetyl-L-homoserine
2.3.1.31
L-Homoserine
Trithionate
2.8.1.5
H2S
2.5.1.47
2.5.1.65
4.4.1.2
2.3.1.46
Reductive carboxylate cycle
Acetate
L-Cysteine
L-Homocysteine
4.4.1.8
Cystathionine
2.5.1.48
O-Succinyl-L-homoserine
Cysteine and methionine metabolism
00920 6/11/12
(c) Kanehisa Laboratories

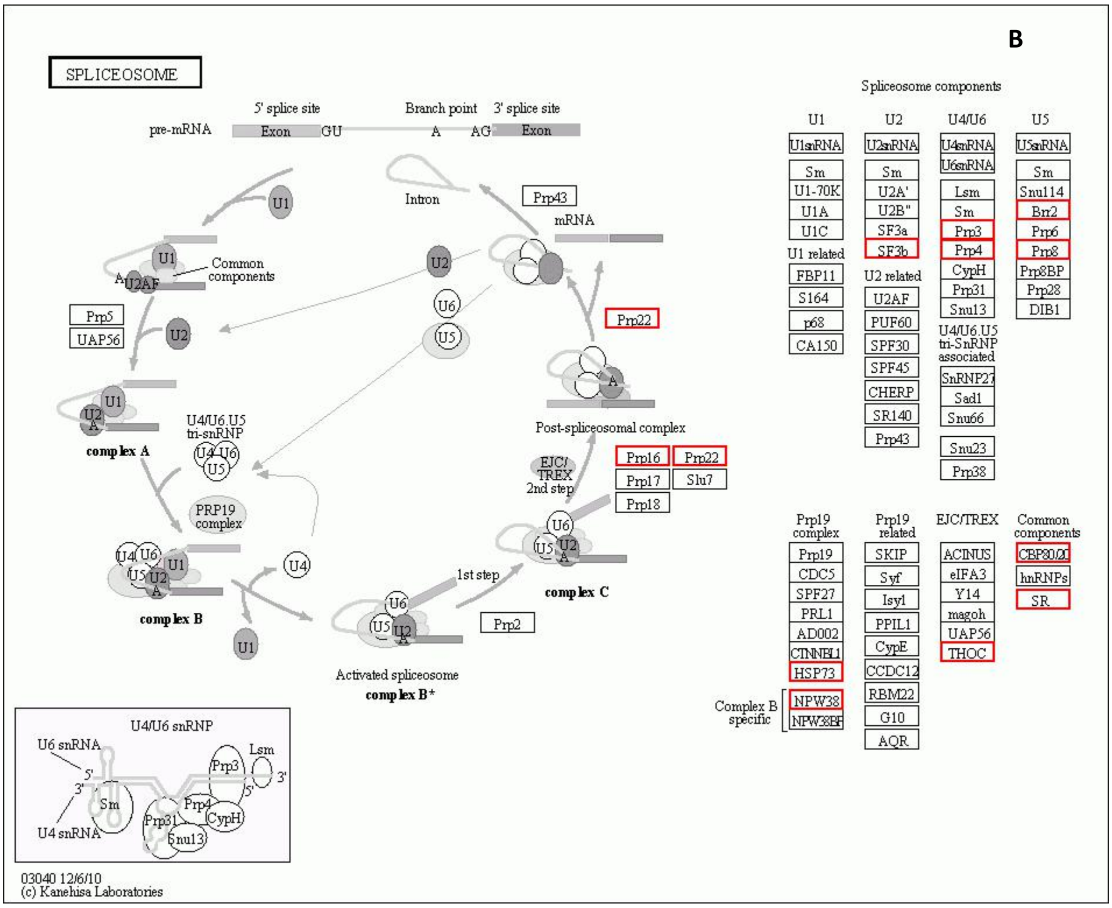
B
SPLICEOSOME
5' splice site
Branch point
3' splice site
pre-mRNA
Exon
GU
A
AG
Exon
Intron
Prp43
mRNA
U1
U2
U6
U5
Common components
U2AF
Prp5
UAP56
Prp22
complex A
U4/U6.U5 tri-snRNP
Post-spliceosomal complex
EJC/ TREX 2nd step
Prp16
Prp22
Prp17
Slu7
Prp18
PRP19 complex
U4
complex B
1st step
complex C
Prp2
Activated spliceosome
complex B*
U4/U6 snRNP
U6 snRNA
U4 snRNA
Lsm
Prp3
Sm
Prp4
CypH
Prp31
Snu13
Spliceosome components
U1
U2
U4/U6
U5
U1snRNA
U2snRNA
U4snRNA
U6snRNA
U5snRNA
Sm
Sm
Sm
U1-70K
U2A'
Lsm
Snu114
U1A
U2B"
Sm
Brr2
U1C
SF3a
Prp3
Prp6
U1 related
SF3b
Prp4
Prp8
FBP11
U2 related
CypH
Prp8BP
S164
U2AF
Prp31
Prp28
p68
PUF60
Snu13
DIB1
CA150
SPF30
U4/U6.U5 tri-SnRNP associated
SPF45
SnRNP27
CHERP
Sad1
SR140
Snu66
Prp43
Snu23
Prp38
Prp19 complex
Prp19 related
EJC/TREX
Common components
Prp19
SKIP
ACINUS
CBP80/20
CDC5
Syf
eIFA3
hnRNPs
SPF27
Isy1
Y14
SR
PRL1
PPIL1
magoh
AD002
CypE
UAP56
CTNNBL1
CCDC12
THOC
HSP73
RBM22
Complex B specific
NPW38
NPW38BP
G10
AQR
03040 12/6/10
(c) Kanehisa Laboratories

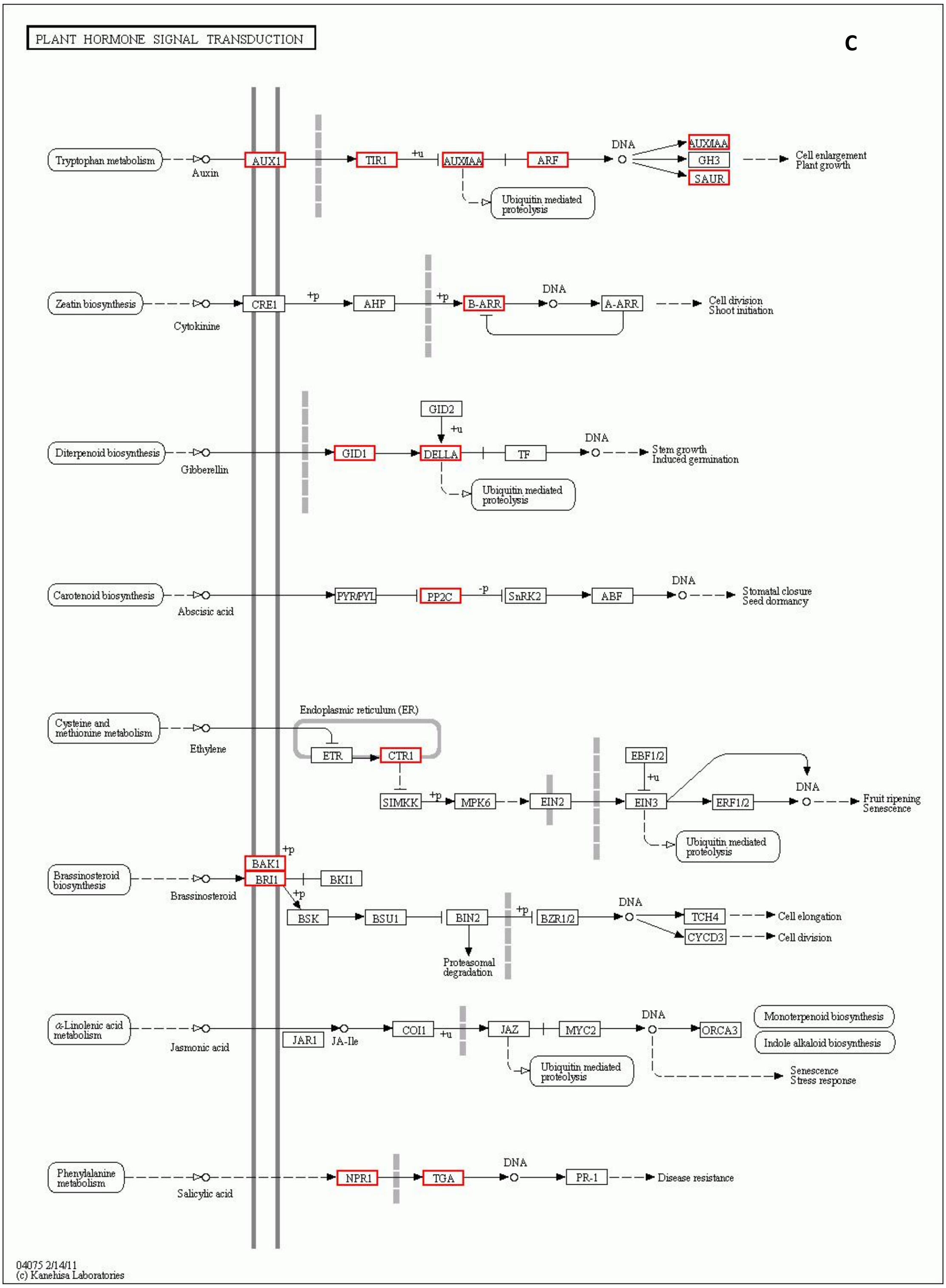

PLANT HORMONE SIGNAL TRANSDUCTION
C
Tryptophan metabolism
Auxin
AUX1
TIR1
+u
AUX/IAA
ARF
DNA
AUX/IAA
GH3
SAUR
Cell enlargement
Plant growth
Ubiquitin mediated proteolysis
Zeatin biosynthesis
Cytokinine
CRE1
+p
AHP
+p
B-ARR
DNA
A-ARR
Cell division
Shoot initiation
GID2
+u
Diterpenoid biosynthesis
Gibberellin
GID1
DELLA
TF
DNA
Stem growth
Induced germination
Ubiquitin mediated proteolysis
Carotenoid biosynthesis
Abscisic acid
PYR/PYL
PP2C
-p
SnRK2
ABF
DNA
Stomatal closure
Seed dormancy
Endoplasmic reticulum (ER)
Cysteine and methionine metabolism
Ethylene
ETR
CTR1
EBF1/2
+u
DNA
SIMKK
+p
MPK6
EIN2
EIN3
ERF1/2
Fruit ripening
Senescence
Ubiquitin mediated proteolysis
+p
BAK1
Brassinosteroid biosynthesis
Brassinosteroid
BRI1
BKI1
+p
BSK
BSU1
BIN2
+p
BZR1/2
DNA
TCH4
Cell elongation
CYCD3
Cell division
Proteasomal degradation
α-Linolenic acid metabolism
Jasmonic acid
JAR1
JA-Ile
COI1
+u
JAZ
MYC2
DNA
ORCA3
Monoterpenoid biosynthesis
Indole alkaloid biosynthesis
Ubiquitin mediated proteolysis
Senescence
Stress response
Phenylalanine metabolism
Salicylic acid
NPR1
TGA
DNA
PR-1
Disease resistance
04075 2/14/11
(c) Kanehisa Laboratories

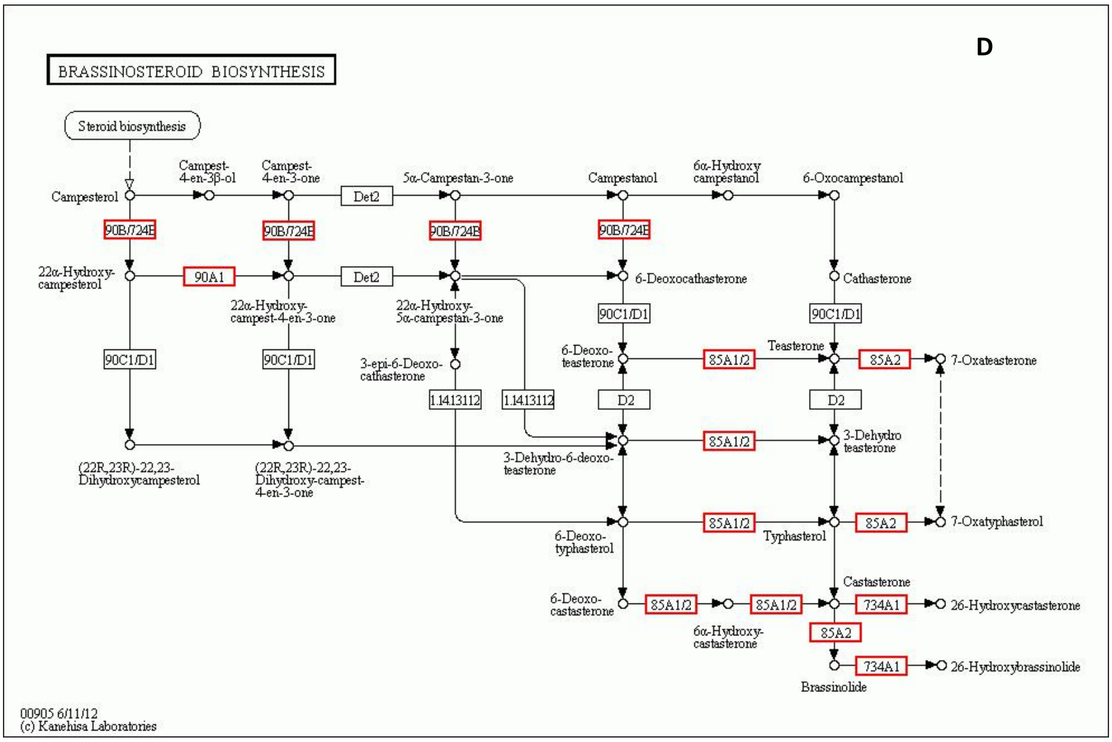

Figure S2. Some metabolic pathway of significant enrichment in the first 20 sites of KEGG.

A represents sulfur metabolism pathway, B represents alternative splicing, C represents plant hormone signal transduction, D represents Brassinosteroid biosynthesis.
